# Atherogenic Index of Plasma Predicts Hyperuricemia in Rural Population: A Cross-Sectional Study from Northeast China

**DOI:** 10.3390/ijerph13090879

**Published:** 2016-09-02

**Authors:** Ye Chang, Yuan Li, Xiaofan Guo, Liang Guo, Yingxian Sun

**Affiliations:** Department of Cardiology, the First Hospital of China Medical University, 155 Nanjing North Street, Heping District, Shenyang 110001, China; chang.ye@stu.xjtu.edu.cn (Y.C.); xi.aohan1989@163.com (Y.L.); guoxiaofan1986@foxmail.com (X.G.); 13654970960@126.com (L.G.)

**Keywords:** atherogenic index of plasma, hyperuricemia, rural population

## Abstract

We aimed to determine the association of atherogenic index of plasma (AIP) with hyperuricemia (HUA) in the rural population of northeast China. This cross-sectional study was conducted in the rural areas of northeast China from January 2012 to August 2013, and the final analysis included data obtained form 5253 men and 6092 women. 1104 participants (9.7%) suffered from HUA. Spearman rank test showed that AIP was positively correlated with uric acid in both sexes (*r* = 0.310 for men and *r* = 0.347 for women, both *p* < 0.001). AIP was classified into three groups: the low (<0.11), the intermediate (0.11–0.21) and the increased (>0.21) risk. The prevalence of HUA increased with AIP. Multivariate logistic regression analysis showed that, compared to the low AIP group, participants in increased AIP group had a 2.536-fold risk for HUA (2.164-fold in male and 2.960-fold in female) after adjustment for covariates. Results of receiver operating characteristic curves showed that the area under the curve (95% confidence intervals) was 0.686 (0.665–0.707) for male and 0.730 (0.706–0.755) for female. We indicated that increased AIP was associated with higher serum uric acid levels and could be identified as an independent risk factor of HUA in the rural population of northeast China.

## 1. Introduction

Hyperuricemia (HUA) is a major public health problem that threatens human health. With the improvement of living standards, high purine, and high protein diet increase, and thus the prevalence of HUA has been increasing in recent decades worldwide [1,2,3,4]. Data from a national survey demonstrated that the prevalence of HUA among Chinese adults in 2009–2010 was 8.4% (9.9% in males and 7.0% in females, respectively) [1]. The prevalence of HUA among US adults in 2007–2008 was 21.2% in male and 21.6% in female, respectively [2]. A nationwide population-based study conducted in Italy indicated that the prevalence of HUA increased from 8.5% in 2005 to 11.9% in 2009 [3]. Data from the Bangkok population showed that 11% of females but an amazing 59% of males had HUA [4]. At the same time, a number of epidemiological studies have reported that HUA could increase the risk of ischemic stroke, acute myocardial infarction, and other cardiovascular events [5,6,7,8] and was a strong predictor of all-cause and cardiovascular disease (CVD) mortality [9,10,11,12,13] in several studies.

Dyslipidemia, identified as an important risk factor for CVD, was accountable for 56% of heart disease and 18% of cases of infarction and it was associated with one third of deaths worldwide [14]. According to The Adult Treatment Panel III (ATPIII) criterion, dyslipidemia was defined as having at least one of high total cholesterol (TC), low high-density lipoprotein cholesterol (HDL), elevated triglycerides (TG), and high low-density lipoprotein cholesterol (LDL) levels. However, compared to these traditional serum lipid variables, studies found that atherogenic index of plasma (AIP) may be a better predictor of CVD risk [15,16]. AIP was calculated as log (TG/HDL) [15]. After the logarithmical transformation, AIP could correct for the lack of normative distribution and demonstrate a correlation with smaller LDL particles and increased fractional esterification rate (FER_HDL_) [15]. Initially, log (TG/HDL) was named AIP because it had a high predictive value for atherosclerosis [17]. Subsequent studies demonstrated that AIP was also significantly correlated with acute coronary events [18], CVD, and its risk factors [15,16,19,20].

Some studies have indicated that elevated serum UA was strongly correlated with increased TC, TG, LDL, and decreased HDL [21,22]. However, the relationship between serum uric acid (UA) and dyslipidemia is so complex that it is not fully elucidated. Furthermore, the association between AIP and serum UA levels is still the subject of much discussion. For this reason, we aimed to determine the association between AIP and UA and assess the capacity of AIP to identify individuals with HUA in the rural populations of northeast China.

## 2. Materials and Methods

### 2.1. Study Population

This study was conducted in Liaoning Province, located in Northeast China. From January 2012 to August 2013, a representative sample of individuals aged ≥35 years was selected to describe the prevalence, incidence, and natural history of cardiovascular risk factors in rural areas of Liaoning Province, which was called Northeast China Rural Cardiovascular Health Study (NCRCHS). The study employed a multi-stage, stratified random cluster-sampling scheme. In the first stage, three counties (Dawa, Zhangwu, and Liaoyang County) were selected randomly from the rural areas of Liaoning province, and in the second stage, one town was randomly selected from each of the three counties. In the third stage, 26 rural villages and 3 towns were randomly selected for inclusion in the study. All eligible permanent residents aged ≥35 years (a total of 14,016 individuals) in each village were invited to participate in the study. Of those, 11,956 participants agreed to participate and completed the present study. The study protocol was approved by the Ethics Committee of China Medical University (Shenyang, China, ethical approved project identification code: 2011-2-2), and all procedures were performed in accordance with good ethical standards.

### 2.2. Data Collection

Written consent was obtained from all participants after they had been informed of the study’s objectives, benefits, and medical procedures, and had signed a confidentiality agreement regarding personal information. Participants who were illiterate completed their Informed Consent with the aid of their proxy. Only participants with a complete set of data for the variables analyzed in the present study were included in the final analysis and individuals who were pregnant or had a malignant tumor or mental disorder were excluded. The final sample size included 11,345 participants (5253 males and 6092 females). Participants in our study belonged to natural population who could be healthy people, diabetics, hypertensive, and participants suffering from cardiovascular diseases or other morbidities.

### 2.3. Lifestyle Factors

Information on covariates, such as age, gender and lifestyle, was collected during a single clinic visit by face-to-face interview by cardiologists and trained nurses using a standard questionnaire. Before the survey was performed, all eligible investigators attended a training session including the purpose of this study, how to administer the questionnaire, the standard method of measurement, the importance of standardization, and the study procedures. A strict test was used for evaluation after this training, and only those who scored perfectly on the test could become investigators. During data collection, study monitors and auditors provided further instruction and support as needed. Each study participant’s race was classified as either Han or other (which included ethnic minorities such as Mongol and Manchu). Family income was classified as ≤5000, 5000–20,000, and >20,000 CNY/year. Educational level was categorized as low (no schooling, incomplete primary education, and primary education), middle (three or four years of secondary education), and high (college and university education). The individuals were asked whether or not they were currently smoking or drinking.

### 2.4. Blood Pressure Measurements

According to the American Heart Association protocol, blood pressure (BP) was measured three times at 2-min intervals after at least 5 min of rest using a standardized automatic electronic sphygmomanometer (HEM-907, Omron, Osaka, Japan), which had already been validated according to the British Hypertension Society protocol [23]. The participants were advised to avoid caffeinated beverages and exercise for at least 30 min before the measurement. During the measurement, the participants were seated with the arm supported at the level of the heart. The mean of three BP measures was calculated and used in all analyses.

### 2.5. Anthropometric Measurements

Weight and height were measured to the nearest 0.1 kg and 0.1 cm, respectively, with participants dressed in light weight clothing and without shoes. Waist circumference (WC) was measured at the umbilicus level using a non-elastic tape (to the nearest 0.1 cm), at the end of normal expiration with the participants standing. Body mass index (BMI) was calculated as weight in kilograms divided by the square of the height in meters.

### 2.6. Serum Analysis

Fasting blood samples were collected in the morning after at least 12 h of fasting for all participants. Blood samples were obtained from an antecubital vein into vacutainer tubes containing EDTA. UA, fasting plasma glucose (FPG), TC, LDL, HDL-C, TG, and other routine blood biochemical indexes were analyzed enzymatically on an autoanalyzer (SYSMEX, Kobe, Japan). All laboratory equipment was calibrated, and blinded duplicate samples were used.

### 2.7. Definition of Dyslipidemia

High TC was defined as TC ≥ 6.21 mmol/L (240 mg/dL). Low HDL was defined as HDL < 1.03 mmol/L (40 mg/dL). High LDL was de fined as LDL ≥ 4.16 mmol/L (160 mg/dL). High TG was defined as ≥ 2.26 mmol/L (200 mg/dL). Dyslipidemia was defined as having at least one of high TC, high LDL, low HDL, and high TG according to the ATP III criteria [14].

### 2.8. Definition of AIP

According to previous studies, AIP was calculated as log (TG/HDL) and was classified into three groups: low (<0.11), intermediate (0.11–0.21), and increased (>0.21) risk [15,24,25].

### 2.9. Definition of Hyperuricemia

Male subjects with SUA ≥ 420.0 μmol/L (7.0 mg/dL) and female subjects ≥ 342.0 μmol/L (5.7 mg/dL) were diagnosed with HUA, according to the national health and nutrition examination survey (NHANES) [2].

### 2.10. Statistical Analyses

The general characteristics of normal subjects and subjects with HUA were compared using *t* test for continuous variables (shown as mean ± standard deviation) and χ^2^ for categorical variables (shown as %). The relationship between uric acid and AIP was examined using Spearman rank test. The prevalence of HUA according to AIP categories was calculated. The odds ratios (ORs) and their 95% confidence intervals (CIs) for the presence of hyperuricemia were estimated by logistic regression analysis with adjustments made for age, race, family income, education, smoking and alcohol status, BMI, WC, systolic blood pressure (SBP), diastolic blood pressure (DBP), FPG, LDL, and TC. We used the area under the receiver-operating characteristic curve (AUC) and 95% CIs to assess the discriminatory power of AIP to predict the risk for HUA. All the statistical analyses were performed using SPSS Statistics for Windows, Version 22.0 (SPSS, Chicago, IL, USA), and *p*-values < 0.05 were considered statistically significant.

## 3. Results

A total of 11,956 permanent residents aged ≥35 years entered the present study; 11,345 of them had complete baseline data and were enrolled in the final analysis. The average age of the residents was 53.8 ± 10.6 and 5253 (46.3%) of them were male. Among them, 1104 (9.7%) were diagnosed with HUA.

As shown in Table 1, participants diagnosed with HUA tended to be older and male. They had a higher value of BMI and WC. Meanwhile, SBP, DBP, FPG, LDL, TC, TG, AIP, and SUA levels were significantly higher in HUA group and HDL showed the opposite trend. Overall education level and family income in the rural were low and showed no difference in the two groups. Compared to some ethnic minorities, the Han nationality suffered more easily from HUA. Additionally, subjects with HUA were more likely to be smokers or drinkers.

**Figure 1:**
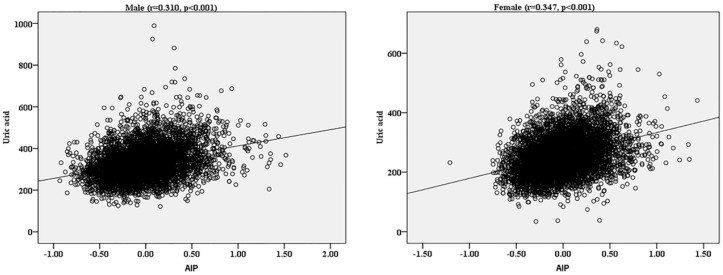
Spearman rank correlation of AIP with UA by sexes. AIP: atherogenic index of plasma; UA, uric acid.

Figure 1 shows the results of Spearman rank test of AIP with UA. The results showed that AIP was positively correlated with UA in both sexes (*r* = 0.310 for men and *r* = 0.347 for women, both *p* < 0.001). The scatterplot also shows a positive correlation between AIP and UA.

**Figure 2:**
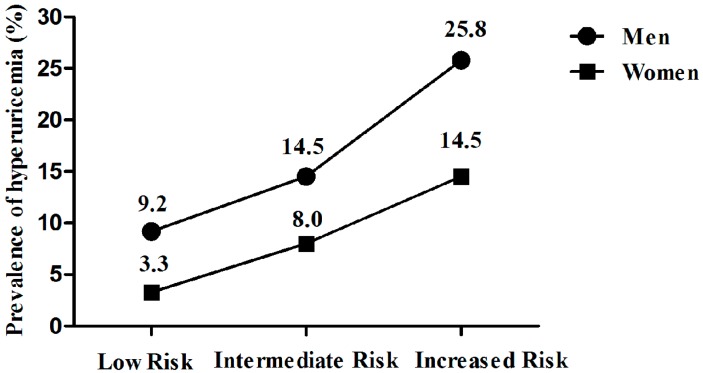
The prevalence of hyperuricemia in the three groups of AIP classified by sexes.

Then participants were classified into three groups according to AIP: low (<0.11), intermediate (0.11–0.21), and increased (>0.21) risk. Figure 2 showed the prevalence of HUA in the three groups of AIP. In males, the prevalence of HUA increased from 9.2% in the low AIP group to 25.8% in the increased AIP group; while in females, the prevalence of HUA increased from 3.3% in the low AIP group to 14.5% in the increased AIP group.

Table 2 showed the multivariate-adjusted ORs (and 95% CIs) of the presence of HUA for AIP. Overall, the risk of suffering from HUA increased with AIP increasing, after adjustment for age, race, family income, education, smoking, and alcohol status, BMI, WC, SBP, DBP, FPG, LDL, and TC. Taking the low AIP group as reference, participants in increased AIP group had a 2.164-fold change risk for HUA in male and a 2.960-fold change risk for HUA in females.

**Figure 3:**
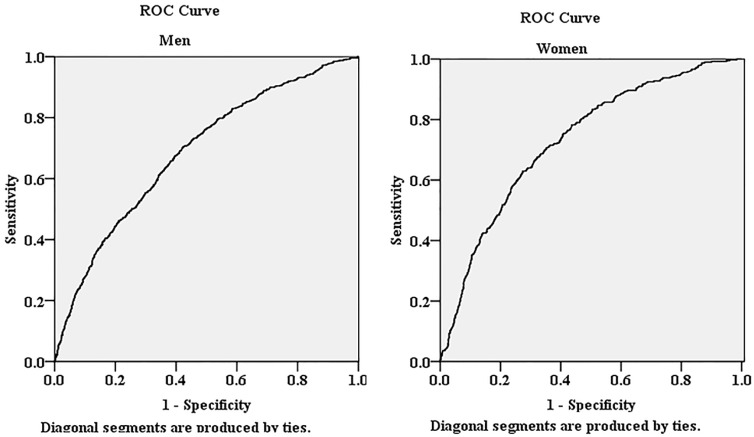
The area under the receiver operating characteristic (ROC) curves of AIP for the presence of HUA in both sexes.

Figure 3 showed the AUCs (and 95% CIs) of AIP in the prediction of HUA. The AUC was 0.686 (95% CIs: 0.665–0.707) for male and 0.730 (95% CIs: 0.706–0.755) for female.

## 4. Discussion

In this cross-sectional study, we mainly found that AIP was positively correlated with serum UA levels and AIP was increased in subjects with HUA in rural population of northeast China.

The results from the Chinese National and Health Survey in 2002 showed that the prevalence of dyslipidemia in the Chinese population was 18.6% [26]. Our results showed that the prevalence of dyslipidemia was 36.4% (data not shown) among the rural population in northeast China. Several lines of studies have demonstrated that dyslipidemia was identified as an important risk factor for CVD [14,15,16]. Compared to traditional pro-atherogenic lipid profile characterized by high TC, high TG, high LDL, and low HDL, AIP could correct for the lack of normative distribution and demonstrate a correlation with smaller LDL particles [15]. Studies found AIP had a high predictive value for atherosclerosis [17], acute coronary events [18], CVD and its risk factors [19,20], and even better than the traditional pro-atherogenic lipid profile [15,16].

A national survey from China in 2009–2010 demonstrated that the prevalence of HUA among Chinese adults was 8.4% [1]. Our study showed that the prevalence of dyslipidemia was 9.7% (calculated according to the baseline data) among the rural population in northeast China. Similar to dyslipidemia, HUA was also an important risk factor for CVD [5,6,7,8,9,10,11,12,13]. Akbas EM et al. found that the AIP level was higher in the HUA group compared to normal UA group and AIP could be identified as independent predictor of HUA in diabetic patients [25]. Consistently, we also found that AIP was positively correlated with serum UA levels and participants in increased AIP group had a 2.536-fold change risk for HUA (2.164-fold for male and 2.960-fold for female, respectively) after adjustment for age, sex, race, family income, education, smoking, and alcohol status, BMI, WC, SBP, DBP, FPG, LDL, and TC. The mechanistic explanation for the links between HUA and AIP might be that they were virtually different phenotypes of insulin resistance [25,27]. On the one hand, high TG could influence the renal function, and thus reduce renal excretion of UA and increase the serum UA level [28,29,30]; on the other hand, decreased HDL was significant correlation with increased serum UA level [31,32]. Taked together, increased AIP (increased TG/decreased HDL) was positively associated with HUA [25,33]. Interestingly, previous studies demonstrated that genetic factors played an important role in regulating lipid profiles and UA. Vuorinen-Markkola H. et al. reported that NADPH was involved in the synthesis of TG, and at the same time could result in increased UA production [34]. This overlap may partly explain why HUA and dyslipidemia often coexist.

### Limitations

Some limitations should also be considered in light of these results. Firstly, this was a cross-sectional study and could not definitively provide a causal relationship between health factors/behaviors and HUA. Second, participants enrolled in our study were from rural areas of China, thus, the results should be interpreted cautiously. Third, potential confounders, especially those could affect SUA levels, such as lifestyle and dietary habits, were not considered. Therefore, residual confounding may exist in the multivariable logistic regression analyses.

## 5. Conclusions

We indicated that increased AIP was strongly associated with higher serum UA levels and could be identified as an independent risk factor of HUA in the rural population of northeast China. As previous studies indicated, lipid profiles were so strongly correlated with UA that we could not just take dyslipidemia into account and leave HUA behind.

## Figures and Tables

**Table 1:** Baseline characteristics of study population.

Variables	Total (*n* = 11,345)	Normal (*n* = 10,241)	Hyperuricemia (*n* = 1104)	*p*-Value
Age (years)	53.8 ± 10.6	53.7 ± 10.9	54.7 ± 11.0	<0.01
Man (%)	5253 (46.3)	4535 (44.3)	718 (65.0)	<0.001
Education Level	-	-	-	0.337
Low	5760 (50.8)	5101 (49.8)	551 (49.9)	-
Middle	4539 (38.4)	4195 (41.0)	428 (38.8)	-
High	1046 (9.2)	945 (9.2)	125 (11.3)	-
Family Income (CNY/year)	-	-	-	0.801
≤5000	1607 (14.2)	1255 (12.3)	149 (13.5)	-
5000–20,000	6060 (53.4)	5610 (54.8)	583 (52.8)	-
>20,000	3678 (32.4)	3376 (32.9)	372 (33.7)	-
Smokers (%)	4007 (35.3)	3569 (34.9)	438 (39.5)	<0.001
Drinkers (%)	2565 (22.6)	2190 (21.4)	375 (34.0)	<0.001
Han (%)	10,759 (94.8)	9697 (94.7)	1062 (96.2)	<0.05
Others ^a^ (%)	586 (5.2)	544 (5.3)	42 (3.8)	-
BMI (kg/m^2^)	24.8 ± 3.7	24.6 ± 3.6	26.5 ± 3.9	<0.001
WC (cm)	82.4 ± 9.8	81.7 ± 9.6	88.4 ± 9.8	<0.001
Serum Indicators	-	-	-	-
Uric Acid (mg/dL)	291.9 ± 84.8	273.9 ± 64.5	458.5 ± 68.0	<0.001
SBP (mmHg)	141.7 ± 23.4	140.3 ± 23.0	153.2 ± 24.0	<0.001
DBP (mmHg)	82.0 ± 11.8	81.6 ± 11.6	85.5 ± 12.5	<0.001
LDL (mmol/L)	2.9 ± 0.8	2.9 ± 0.8	3.1 ± 0.9	<0.001
HDL (mmol/L)	1.4 ± 0.4	1.4 ± 0.4	1.3 ± 0.4	<0.001
TG (mmol/L)	1.6 ± 1.5	1.5 ± 1.3	2.5 ± 2.5	<0.001
TC (mmol/L)	5.2 ± 1.1	5.2 ± 1.1	5.6 ± 1.2	<0.001
FPG (mmol/L)	5.9 ± 1.6	5.9 ± 1.7	6.1 ± 1.5	<0.001
AIP	−0.009 ± 0.32	−0.03 ± 0.31	0.20 ± 0.33	<0.001

Abbreviations: AIP, atherogenic index of plasma; BMI, body mass index; CNY, China Yuan (1 CNY = 0.154 dollar); DBP, diastolic blood pressure; FPG, fasting plasma glucose; HDL, high-density lipoprotein; LDL, low-density lipoprotein cholesterol; SBP, systolic blood pressure; TC, total cholesterol; WC, waist circumference; ^a^ Including some ethnic minorities in China, such as Mongol and Manchu.

**Table 2:** Multivariable logistic regression analyses on the association between the three groups of AIP and HUA ^a^.

**Men**	**Odds Ratios**	**95% Confidence Intervals**	***p* Value**
1 (Referenced Group)	1	-	-
2	1.315	0.989, 1.748	0.059
3	2.164	1.782, 2.628	<0.001
**Women**	**Odds Ratios**	**95% Confidence Intervals**	***p* Value**
1 (Referenced Group)	1	-	-
2	1.849	1.305, 2.620	<0.001
3	2.96	2.311, 3.792	<0.001

^a^ adjusted for age, race, family income, education, smoking and alcohol status, BMI, WC, SBP, DBP, FPG, LDL, and TC.
